# Phytosiderophores determine thresholds for iron and zinc accumulation in biofortified rice endosperm while inhibiting the accumulation of cadmium

**DOI:** 10.1093/jxb/erx304

**Published:** 2017-09-28

**Authors:** Raviraj Banakar, Ana Alvarez Fernandez, Pablo Díaz-Benito, Javier Abadia, Teresa Capell, Paul Christou

**Affiliations:** 1 Departament de Producció Vegetal i Ciència Forestal, Universitat de Lleida-Agrotecnio Center Lleida, Spain; 2 Department of Plant Nutrition, Estación Experimental de Aula Dei, Consejo Superior de Investigaciones Científicas (CSIC), Zaragoza, Spain; 3 ICREA, Catalan Institute for Research and Advanced Studies, Passeig Lluís Companys, Barcelona, Spain

**Keywords:** Cadmium, 2′-deoxymugenic acid (DMA), iron and zinc homeostasis, metal transporters, nicotianamine, rice

## Abstract

Nicotianamine (NA) and 2′-deoxymugenic acid (DMA) are metal-chelating ligands that promote the accumulation of metals in rice endosperm, but it is unclear how these phytosiderophores regulate the levels of different metals and limit their accumulation. In this study, transgenic rice plants producing high levels of NA and DMA accumulated up to 4-fold more iron (Fe) and 2-fold more zinc (Zn) in the endosperm compared with wild-type plants. The distribution of Fe and Zn in vegetative tissues suggested that both metals are sequestered as a buffering mechanism to avoid overloading the seeds. The buffering mechanism involves the modulation of genes encoding metal transporters in the roots and aboveground vegetative tissues. As well as accumulating more Fe and Zn, the endosperm of the transgenic plants accumulated less cadmium (Cd), suggesting that higher levels of Fe and Zn competitively inhibit Cd accumulation. Our data show that although there is a strict upper limit for Fe (~22.5 µg g^−1^ dry weight) and Zn (~84 µg g^−1^ dry weight) accumulation in the endosperm, the careful selection of strategies to increase endosperm loading with essential minerals can also limit the accumulation of toxic metals such as Cd, thus further increasing the nutritional value of rice.

## Teaser

We unravel the mechanisms that set upper limits for iron and zinc accumulation in rice endosperm while inhibiting the accumulation of cadmium.

## Introduction

Iron (Fe) and zinc (Zn) are essential metals in plants (Kobayashi *et al.*, 2010; Sinclair and Krämer, 2012). Fe mediates electron transport during photosynthesis and respiration (Connolly and Guerinot, 2002), and both metals act as cofactors for enzymes and regulatory proteins (Sinclair and Krämer, 2012). However, both metals are toxic at high concentrations, and plants have therefore evolved homeostatic mechanisms to regulate their accumulation (Sinclair and Krämer, 2012; Anjum *et al.*, 2015).

Rice (*Oryza sativa*) has two Fe acquisition strategies: the direct uptake of Fe^2+^ (strategy I) and the uptake of Fe^3+^ complexes following the secretion of metal-chelating phytosiderophores (strategy II). In strategy I, Fe^2+^ is taken up into the root epidermis by the membrane-bound Fe-regulated transporters OsIRT1 and OsIRT2, which also transport Zn^2+^ (Vert *et al.*, 2002; Lee and An, 2009). Once inside the plant, Fe^2+^ and Zn^2+^ form complexes with nicotianamine (NA) that are translocated and remobilized internally for seed loading (Cheng *et al.*, 2007; Ishimaru *et al.*, 2010; Nishiyama *et al.*, 2012; Hazama *et al.*, 2015). The Fe-regulated ZIP family transporter proteins OsZIP1–4 and the heavy metal ATPase family transporter OsHMA2 can also transport either Fe^2+^ or Zn^2+^ (Ishimaru *et al.*, 2005; Yamaji *et al.*, 2013). In strategy II, Fe^3+^ in the soil is chelated by the phytosiderophore 2′-deoxymugenic acid (DMA), which is secreted into the rhizosphere by the membrane-bound transporter OsTOM1 (Ma *et al.*, 1999; Nozoye *et al.*, 2011). Fe^3+^–DMA complexes are then taken up into the roots by OsYSL15 and internally transported by OsYSL18 for seed loading (Aoyama *et al.*, 2009). DMA can also bind Zn^2+^, and Zn^2+^–DMA complexes are favored over free Zn^2+^ for internal transport and seed loading (Suzuki *et al.*, 2008).

NA and DMA are involved in metal homeostasis (Masuda *et al.*, 2012; Banakar *et al.*, 2017) and increasing the amount of NA or DMA in the plant increases the accumulation of Fe and Zn in the seeds (Masuda *et al.*, 2009, 2012; Johnson *et al.*, 2011). NA and DMA are synthesized from *S*-adenosylmethionine (SAM) in three steps involving nicotianamine synthase (NAS), nicotianamine aminotransferase (NAAT) and DMA synthase (DMAS) (Ma *et al.*, 1999; Banakar *et al.*, 2017). The disruption of NAAT blocks the pathway and causes NA to accumulate at higher levels than DMA, whereas the overexpression of NAAT increases flux through the pathway and causes DMA to accumulate at higher levels than NA, but both strategies promote Fe and Zn uptake, mobilization and seed loading (Cheng *et al.*, 2007; Johnson *et al.*, 2011; Masuda *et al.*, 2012).

The homeostatic control of Fe and Zn uptake and redistribution in rice maintains the metal content of wild-type rice endosperm within the ranges 2–5 μg Fe g^−1^ dry weight (DW) and 15–20 μg Zn g^−1^ DW (Yang *et al.*, 1998; Sperotto *et al.*, 2012*a*). Rice is a staple crop for more than 50% of the global population (Yuan *et al.*, 2011; Berman *et al.*, 2013; Pérez-Massot *et al.*, 2013) and insufficient levels of Fe and Zn in the endosperm cause deficiency diseases in populations relying on rice as a principal source of calories (Gómez-Galera *et al.*, 2010; Sperotto *et al.*, 2013). More than 2 billion people suffer from Fe deficiency anemia and/or Zn deficiency (Slamet-Loedin *et al.*, 2015). Rice grown on contaminated soils also causes cadmium (Cd) toxicity (Ogawa *et al.*, 2004; Aziz *et al.*, 2015). Therefore, biofortification strategies that enhance Fe and Zn levels in rice endosperm should do so without also encouraging the accumulation of Cd (Clemens *et al.*, 2013; Slamet-Loedin *et al.*, 2015). Although modulating the NA/DMA pathway can increase the accumulation of Fe and Zn in the endosperm, the maximum levels reported are 14–16 µg Fe g^−1^ DW (4× wild-type levels) and 75 µg Zn g^−1^ DW (2× wild-type levels) (Johnson *et al.*, 2011). This suggests there is an upper limit for Fe and Zn accumulation in the endosperm at which metal homeostasis acts to prevent the seed becoming overloaded with these metals, but the underlying mechanisms are largely unknown.

We generated transgenic rice lines co-expressing NAS and NAAT on the basis that these should ectopically produce higher levels of NA and DMA, promoting the accumulation of Fe and Zn in the endosperm and allowing us to investigate the mechanisms responsible for Fe/Zn accumulation threshold in rice endosperm. We grew the transgenic plants in nutrient solutions containing different amounts of Fe and monitored the accumulation of Fe and Zn in the seeds. We also measured the expression of genes encoding metal transporters in vegetative tissues to determine whether metal homeostasis involved the modulation of transporter availability. Finally, we measured the Cd content of the seed to determine whether the accumulation of mineral nutrients could be uncoupled from the accumulation of toxic heavy metals. Our findings provide evidence for mechanisms that limit Fe and Zn accumulation in the endosperm and support the development of biofortification strategies that enhance the accumulation of essential minerals in the endosperm while excluding toxic metals.

## Materials and methods

### Gene cloning

The *OsNAS1* (GenBank: AB021746.2) and *HvNAATb* (GenBank: AB005788.1) cDNAs were cloned from the roots of 2-week-old rice (*Oryza sativa* L cv. EYI 105) and barley (*Hordeum vulgare* L. cv. Ordalie) plants grown *in vitro* on Murashige and Skoog (MS) medium without Fe (Murashige and Skoog, 1962). Total RNA was extracted using the RNeasy Plant Mini Kit (Qiagen, Hilden, Germany) and 1 µg of total RNA was reverse transcribed using the Omniscript RT Kit (Qiagen). The full-size cDNAs for *OsNAS1* (999 bp) and *HvNAATb* (1656 bp) were ampliﬁed by PCR using the primer combinations listed in Supplementary Table S1 at *JXB* online. After sequence verification, both cDNAs were transferred to the expression vector pAL76 (Christensen and Quail, 1996), containing the maize *ubiquitin-1* promoter plus ﬁrst intron and *nos* transcriptional terminator. A separate vector provided the *hpt* selectable marker (Christou and Ford, 1995).

### Generation and analysis of transgenic rice plants

Rice seed-derived embryos (*Oryza sativa* L. cv EYI 105) were co-transformed with *OsNAS1*, *HvNAATB* and *hpt* as previously described (Christou *et al.*, 1991; Sudhakar *et al.*, 1998; Valdez *et al.*, 1998). Transgenic T_0_ plants co-expressing *OsNAS1* and *HvNAATb* were identified by RNA blot analysis using *OsNAS1* and *HvNAATb* probes (Banakar *et al.*, 2017). The plants were self-pollinated to generate T_1_ seeds, which were germinated on half-strength MS medium supplemented with 50 mg l^−1^ hygromycin (wild-type seeds were germinated on the same medium without hygromycin). After 5 d, seedlings were transferred to separate trays and maintained as previously described (Banakar *et al.*, 2017). Roots and leaves from 1-month-old seedlings were harvested to measure the levels of NA, DMA and metals. Seeds were harvested from wild-type and transgenic lines (T_2_ generation) for the same purpose.

### Iron uptake studies

Wild-type seeds and T_2_ seeds from transgenic rice lines EYI-9, EYI-89, and EYI-98 were germinated as above. After 5 d, the seedlings were transferred to independent floating trays and watered for 3 weeks with nutrient solution (Kobayashi *et al.*, 2005) containing 100 µM FeCl_3_. Ten seedlings of equivalent height from each transgenic line (and wild-type controls) were then transferred to independent trays with nutrient solution containing 100 µM FeCl_3_ (normal feeding), 200 µM FeCl_3_ (double Fe regime), or 300 µM FeCl_3_ (triple Fe regime) in isolation. Fresh nutrient solution was provided every other day and the pH of the solution was adjusted to 5.3 with 0.1 M KOH. Every week, the nutrient solution was completely replaced. Plants were maintained as above until the seeds had matured. Samples were collected from 10 plants per group and pooled as roots, culm, middle leaf, flag leaf and T_3_ seeds for the analysis of metal content.

### Cadmium uptake studies

Wild-type seeds and T_3_ transgenic seeds were germinated and maintained in nutrient solution supplemented with 100 µM FeCl_3_ as above. Fifteen seedlings of equivalent height from each transgenic line (and wild-type controls) were then transferred to independent trays of nutrient solution containing 10 μM CdCl_2_ or an equivalent control, and were grown in isolation until seed harvesting.

### Analysis of metal and phytosiderophore levels

The metal content of the roots, leaves and seeds was determined in diluted samples by inductively coupled plasma mass spectrometry (ICP-MS) using an Agilent 7700X instrument (Agilent Technologies, Santa Clara, CA, USA) as previously described (Banakar *et al.*, 2017). NA and DMA levels in roots, flag leaf and seeds were determined by HPLC-electrospray ionization (ESI)-time of flight (TOF)-MS (Xuan *et al.*, 2006) with the modifications described by Banakar *et al.* (2017).

### Quantification of endogenous gene expression

Quantitative real-time RT-PCR was carried out on samples of roots, flag leaf and immature seeds as described by Banakar *et al.* (2017) to measure steady-state mRNA levels representing the endogenous genes listed in Supplementary Table S1.

### Statistical analysis

Differences between transgenic and wild-type plants and the differences among Fe feeding regimes were tested by comparison of means using Student’s *t*-test (*P*<0.05).

## Results

### Rice plants co-expressing NAS and NAAT produce greater quantities of phytosiderophores and accumulate more Fe and Zn in the seeds than wild-type plants

**Fig. 1.**
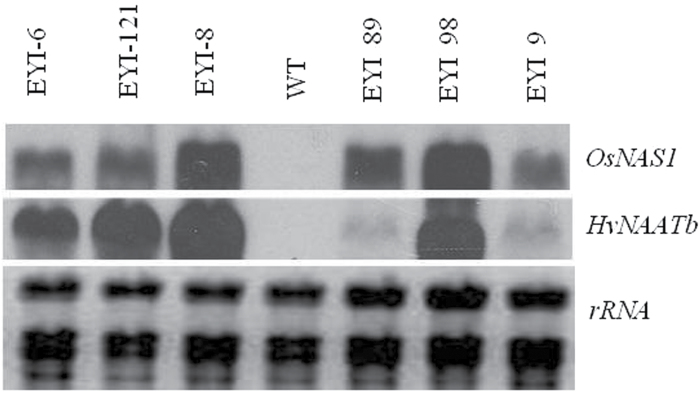
Representative RNA blot analysis showing transgene expression in the leaf tissue of wild-type (WT) and six representative independent T_2_ transgenic lines. *HvNAATb*, barley nicotianamine aminotransferase; *OsNAS1*, rice nicotianamine synthase; rRNA, ribosomal RNA. Wild-type and transgenic lines were grown under nutrient-sufficient conditions, and total RNA was isolated from flag leaves at physiological maturity.

Mature seed-derived zygotic rice embryos were bombarded with constructs encoding rice NAS (*OsNAS1*), barley NAAT (*HvNAATb*) and the selectable marker *hpt*, each controlled by a constitutive promoter. RNA blot analysis revealed the co-expression of *OsNAS1* and *HvNAATb* in several independent lines, a representative selection of which is shown in Fig. 1. T_1_ seeds were harvested from the first 10 lines, and the T_1_ plants were self-pollinated. Neither the T_0_ nor the T_1_ plants differed from wild-type plants in terms of growth, development, seed number, or seed weight. The unpolished T_2_ seeds were analysed by ICP-MS to determine the Fe and Zn content (see Supplementary Table S2), revealing levels of 22–57 µg Fe g^−1^ DW (1.4–3.7 times higher than wild-type) and 22–78 µg Zn g^−1^ DW (1.2–4.2 times higher than wild-type). T_2_ seeds from the three lines with the highest levels of Fe/Zn (EYI-9, EYI-89, and EYI-98) were polished and the Fe and Zn levels were determined, revealing endosperm Fe levels of 8, 16, and 10 µg g^−1^ DW, respectively, compared with 4 µg g^−1^ DW in wild-type seeds, and endosperm Zn levels of 36, 42, and 65 µg g^−1^ DW, respectively, compared with 16 µg g^−1^ DW in wild-type seeds. We also measured Fe and Zn levels in the roots and leaves of T_1_ plants to see if the higher levels in seeds reflected an increase in metal uptake and root-to-shoot translocation. The transgenic roots contained 1.2-fold and 2.2-fold higher levels of Fe and Zn, respectively, compared with wild-type roots, and the transgenic leaves contained 2.2-fold and 2.3-fold higher levels of Fe and Zn, respectively, compared with wild-type leaves (see Supplementary Fig. S1). These results suggest that the co-expression of NAS1 and NAAT in rice enhances Fe and Zn uptake, root-to-shoot translocation, and seed loading.

**Fig. 2.**
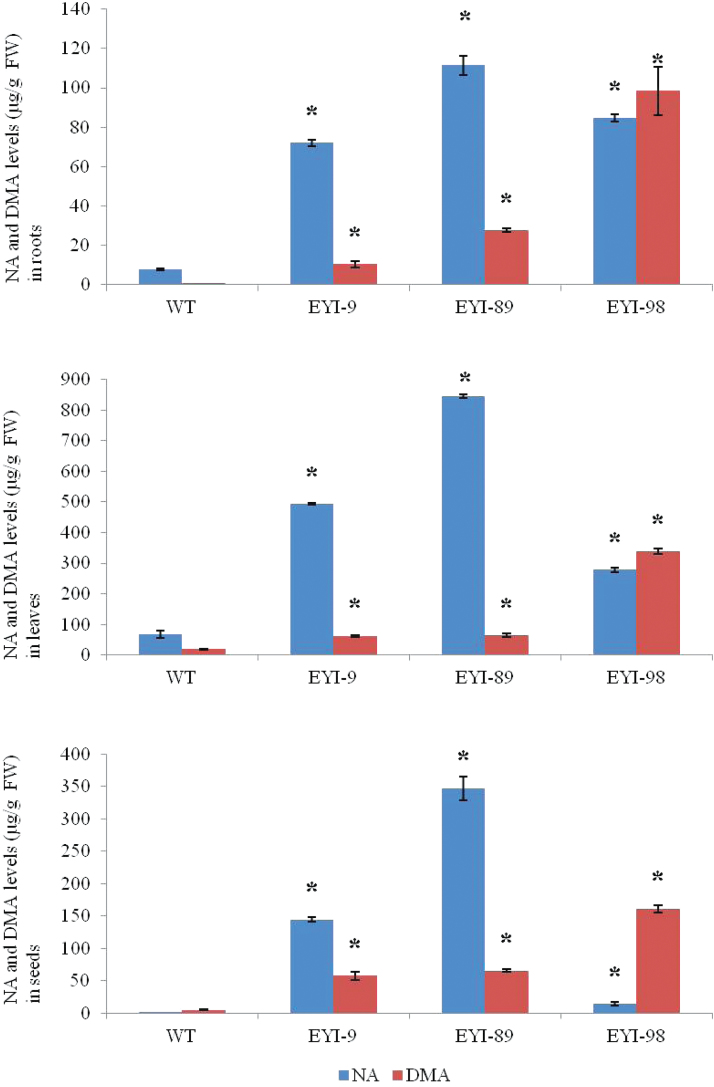
Nicotianamine (NA) and 2′-deoxymugenic acid (DMA) levels (µg g^−1^ FW) in roots, leaves, and unpolished seeds of wild-type (WT) rice and three different T_2_ transgenic lines co-expressing *OsNAS1* and *HvNAATb* (EYI-9, EYI-89, and EYI-98). FW, fresh weight. Asterisks indicate a statistically significant difference between wild-type and transgenic plants as determined by Student’s *t*-test (*P*<0.05; *n*=3). Both the wild-type and transgenic lines were grown under nutrient-sufficient conditions and seeds were harvested at physiological maturity.

Extracts of T_1_ roots and leaves and T_2_ seeds from the same lines were analysed by HPLC-ESI-TOF-MS to determine the levels of NA and DMA (Fig. 2). Transgenic lines accumulated 14-fold, 12-fold, and 160-fold more NA in the roots, leaves, and seeds, respectively, compared with wild-type plants. Similarly, the transgenic lines accumulated 127-fold, 17-fold, and 29-fold more DMA in the roots, leaves, and seeds, respectively, compared with wild-type plants. The NA/DMA ratios differed among the three lines, with EYI-9 and EYI-89 producing more NA than DMA (*P*<0.05; *n*=3) and EYI-98 producing more DMA than NA (*P*<0.05; *n*=3). These data indicate that the increased content of Fe and Zn in the T_2_ transgenic seeds probably reflects the greater abundance of NA and/or DMA, promoting Fe and Zn uptake, root-to-shoot translocation, and seed loading.

### The external Fe supply does not affect Fe and Zn seed loading

**Fig. 3.**
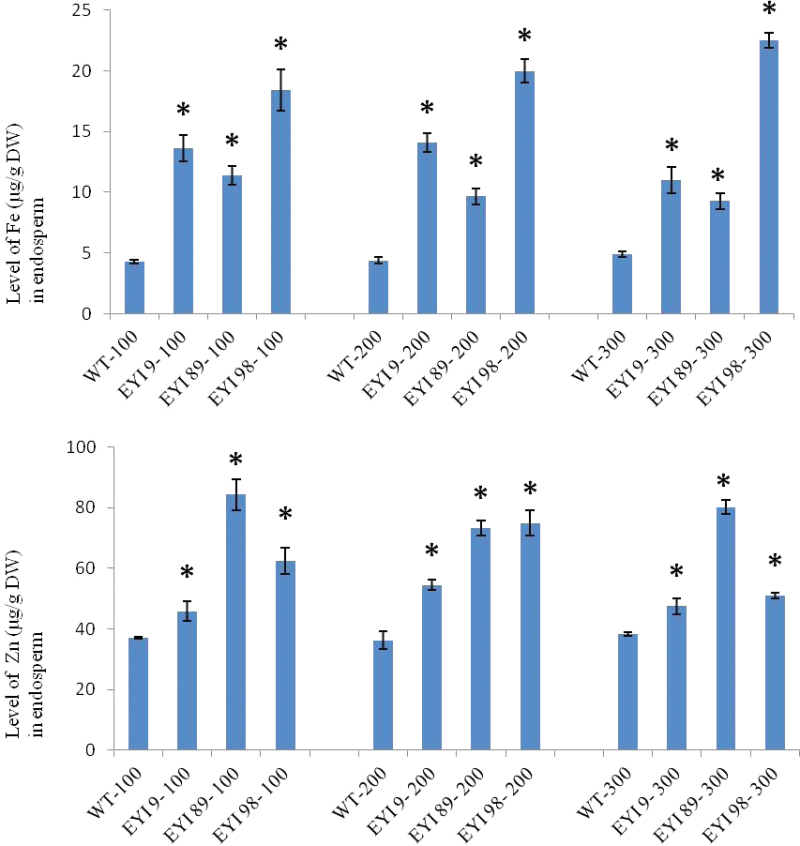
Fe and Zn levels (µg g^−1^ DW) in the endosperm of wild-type (WT) rice and three different T_3_ transgenic lines co-expressing *OsNAS1* and *HvNAATb* (EYI-9, EYI-89, and EYI-98) under 100 (control), 200, and 300 µM Fe treatments. DW, dry weight. Asterisks indicate statistically significant differences between wild-type and transgenic plants under the same Fe feeding regime as determined by Student’s *t*-test (*P*<0.05; *n*=6). Seeds from the wild-type and transgenic lines were harvested at physiological maturity. (This figure is available in color at *JXB* online.)

Next we investigated whether seed loading with Fe and Zn was affected by the abundance of Fe in the environment. We grew T_2_ plants from lines EYI-9, EYI-89, and EYI-98 (plus wild-type controls) under three different feeding regimes, i.e. normal Fe (100 µM), double Fe (200 µM) and triple Fe (300 µM), before measuring the Fe levels in endosperm as above. The T_2_ plants showed no differences in phenotype (growth and development) compared with wild-type plants under any of the Fe feeding regimes (see Supplementary Fig. S2). Under the normal feeding regime, the T_3_ endosperm accumulated 2.6–4.3 times more Fe than wild-type endosperm, whereas the corresponding increments under the double and triple Fe regimes were 2.2–4.5-fold and 1.9–4.6-fold, respectively (Fig. 3). There were no significant differences in terms of Fe accumulation in the endosperm of the wild-type and transgenic lines when comparing the three feeding regimes (*P*>0.05; *n*=6), with the exception of EYI-9, which accumulated significantly less Fe (*P*<0.05; *n*=6) during the 300 µM Fe treatment compared with normal Fe feeding, and EYI-98, which accumulated significantly more Fe (*P*<0.05; *n*=6) during the 300 µM Fe treatment compared with normal Fe feeding (Fig. 3). This confirms the presence of a strict homeostatic mechanism that limits the accumulation of Fe in the endosperm to ~22.5 µg g^−1^ DW regardless of the external Fe supply. The accumulation of Zn in the endosperm also appeared to be governed by an intrinsic threshold independent of the external Fe supply. The T_3_ endosperm accumulated 1.2–2.2 times more Zn than wild-type endosperm under the normal feeding regime. This barely changed under the double Fe regime (1.5–2 times more Zn) or the triple Fe regime (1.2–2 times more Zn) (Fig. 3). There were no significant differences (*P*>0.05; *n*=6) in Zn levels in the endosperm of the wild-type and transgenic lines when comparing the three Fe feeding regimes, with the exception of EYI-98, which accumulated significantly more Zn (*P*<0.05; *n*=6) during the 200 µM Fe treatment but significantly less Zn (*P*<0.05; *n*=6) during the 300 µM Fe treatment, in each case compared with normal Fe feeding (Fig. 3). These data indicate that the external supply of Fe does not alter the threshold level of Zn in the endosperm, which was ~84 µg Zn g^−1^ DW.

### The uptake, translocation, and remobilization of Fe and Zn are influenced by the external supply of Fe

**Fig. 4.**
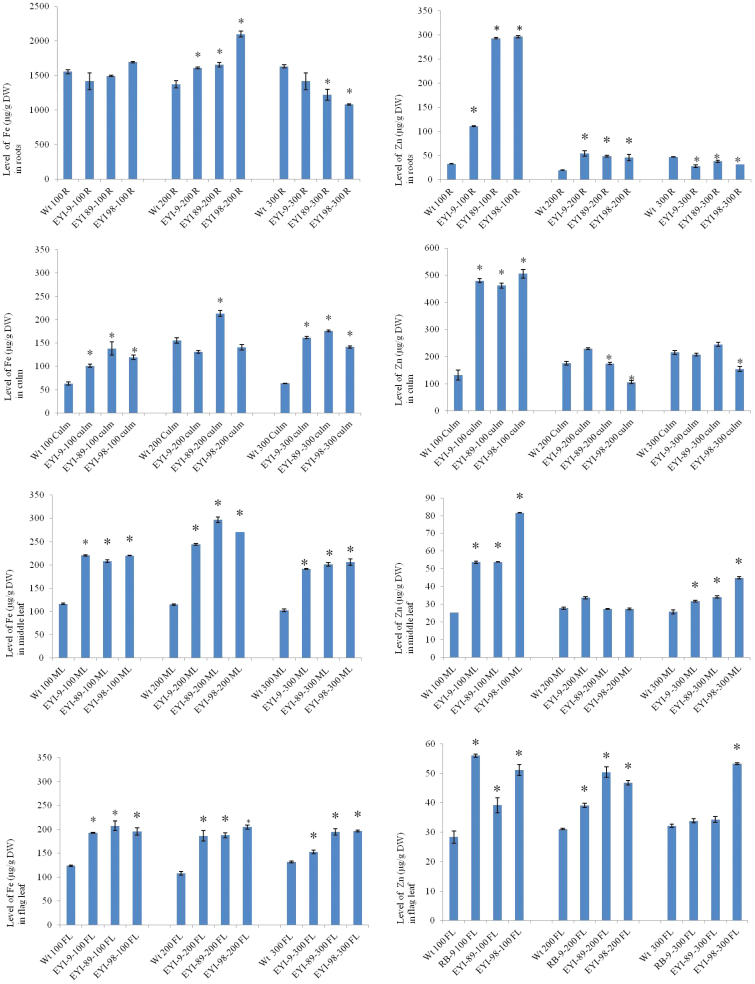
Fe and Zn levels (µg g^−1^ DW) in the roots, culm, middle leaf, and flag leaf of wild-type (WT) rice and three different T_2_ transgenic lines co-expressing *OsNAS1* and *HvNAATb* (EYI-9, EYI-89, and EYI-98) under 100 (control), 200, and 300 µM Fe treatments. DW, dry weight; FL, flag leaf; ML, middle leaf; R, roots. Asterisks indicate statistically significant differences between wild-type and transgenic plants under the same Fe feeding regime as determined by Student’s *t*-test (*P*<0.05; *n*=3). In both the wild-type and transgenic lines, the samples of roots, culm, middle leaf, and flag leaf were harvested at physiological maturity. (This figure is available in color at *JXB* online.)

The negligible impact of external Fe on the accumulation of Fe and Zn in seeds suggests that the excess Fe either remains in the soil or is taken up but sequestered in the vegetative tissues. We therefore measured the levels of Fe and Zn in the roots, culm, middle leaf, and flag leaf of mature plants. Under normal feeding conditions, there was no significant difference in Fe content between the roots of transgenic and wild-type plants (1693 ± 8 *vs* 1555 ± 30 µg g^−1^ DW) (Fig. 4). When the Fe supply was doubled, the roots of the transgenic lines accumulated significantly more Fe than wild-type roots (2101 ± 45 *vs* 1372 ± 55 µg g^−1^ DW), but when the Fe supply was tripled the roots of the transgenic lines accumulated significantly less Fe than wild-type roots (1080 ± 9 *vs* 1631 ± 22 µg g^−1^ DW), suggesting that abundant Fe is initially taken into the roots but an excess triggers feedback that restricts Fe uptake into root cells (Fig. 4). The culms of the transgenic lines accumulated more Fe than the wild-type controls under all three treatments, indicating that Fe root-to-shoot translocation was enhanced in the transgenic plants (Fig. 4). The middle leaf of the transgenic lines accumulated more Fe than wild-type controls under all three treatments, with plants fed on 200 µM Fe accumulating the most (Fig. 4). Although the flag leaves of the transgenic lines accumulated more Fe than the corresponding wild-type plants, there were no significant differences among the three treatments (Fig. 4). These results suggest that although Fe uptake, root-to-shoot translocation, and remobilization are influenced by the external Fe supply, sequestration of Fe in the roots, culm, middle leaf, and flag leaf acts as a buffering mechanism to limit Fe transfer to the endosperm.

The roots of the transgenic lines contained more Zn than wild-type plants under the normal and double Fe feeding regimes, but significantly less Zn under the triple Fe regime, as observed for Fe (Fig. 4). This suggests that higher levels of Fe in the nutrient solution inhibited Zn uptake. Higher levels of Zn were present in the culms of transgenic plants compared with wild-type controls under normal feeding conditions (505 ± 15 *vs* 131 ± 18 µg g^−1^ DW), indicating more efficient Zn root-to-shoot translocation, but there was little difference between the transgenic and wild-type culms at 200 and 300 µM Fe, suggesting that Zn root-to-shoot translocation is suppressed by excess Fe (Fig. 4). The profile in the culm was not replicated precisely in the middle leaf but there was a similar trend: the transgenic lines accumulated more Zn in the middle leaf than wild-type plants under normal feeding (81 ± 0.13 *vs* 25 ± 0.06 µg g^−1^ DW) and to a lesser extent under the triple Fe feeding regime, but there was no significant difference between transgenic and wild-type plants under the double Fe feeding regime (33 ± 0.6 *vs* 27 ± 0.6 µg g^−1^ DW) (Fig. 4). This suggests that the suppression of Zn root-to-shoot translocation also affects Zn mobilization to the middle leaf. The amount of Zn in the flag leaves was generally higher in the transgenic lines than wild-type controls under all three treatments, and across the treatments the transgenic lines generally behaved in a similar manner (Fig. 4). This suggests Zn sequestration in the flag leaf acts as a buffering mechanism to limit Zn loading in the endosperm.

### The external supply of Fe modulates the expression of metal transporter genes in vegetative tissues to limit endosperm loading with Fe and Zn

**Fig. 5.**
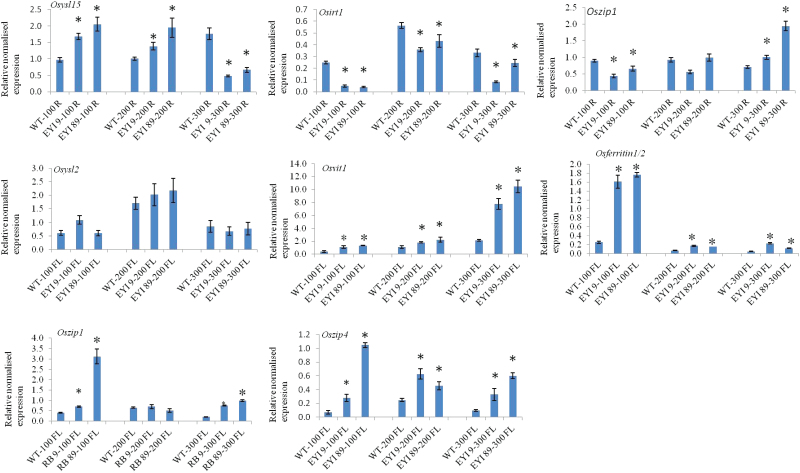
Quantitative real-time PCR analysis of *OsYSL15*, *OsIRT1*, and *OsZIP1* in roots (R) and *OsYSL2*, *OsVIT1*, *OsFERRITIN1*, *OsZIP1*, and *OsZIP4* in flag leaf (FL) under 100 (control), 200, and 300 µM Fe treatments at physiological maturity in wild-type rice and T_3_ transgenic lines co-expressing *OsNAS1* and *HvNAATb* (EYI-9, EYI-89). Each value is the mean of three independent experiments. The *OsYSL5*, *OsIRT1*, *OsZIP1*, *OsZIP4*, *OsVIT1*, *OsYSL2*, and *OsFERRITIN1* mRNA levels were normalized to Os*ACTIN1*. Asterisks indicate statistically significant differences between wild-type and transgenic plants under the same Fe feeding regime as determined by Student’s *t*-test (*P*<0.05; *n*=3). Gene-specific primers are listed in Table S1. (This figure is available in color at *JXB* online.)

Metal transporters play an important role in the uptake, root-to-shoot translocation, sequestration, and remobilization of metals, thus strongly influencing Fe and Zn loading in the endosperm (Banakar *et al.*, 2017). The expression of endogenous metal transporters in transgenic plants expressing NAS and in *OsNAAT1* mutants is regulated by the metal content of different tissues, which in turn is influenced by the absolute and relative levels of NA and DMA (Cheng *et al.*, 2007; Wang *et al.*, 2013). We therefore measured the expression of three metal transporter genes in the roots, encoding the Fe^3+^–DMA transporter OsYSL15, the Fe and Zn transporter OsIRT1, and the Zn transporter OsZIP1. Expression levels were compared in T_3_ transgenic plants (lines EYI-9 and EYI-89) and wild-type controls. We found that *OsYSL15* was upregulated in the roots of the transgenic lines compared with wild-type plants under the normal and double Fe feeding regimes, but was downregulated under the triple Fe feeding regime (Fig. 5). *OsIRT1* was downregulated in the transgenic lines compared with wild-type plants under all three treatments, with the lowest expression level observed under the normal feeding regime (Fig. 5). *OsIRT1* expression in the transgenic lines was higher under the double Fe feeding regime than the triple Fe feeding regime (Fig. 5), resulting in a significant difference (*P*<0.05; *n*=3) in *OsIRT1* expression between transgenic plants under the double and triple Fe feeding regimes *versus* wild-type plants under the normal feeding regime. *OsZIP1* was downregulated in the transgenic lines during normal Fe feeding, there was no significant difference under the double Fe feeding regime, and it was upregulated in the transgenic plants under the triple Fe feeding regime (Fig. 5). These data suggest that the genes for all three transporters are modulated by changes in Fe and Zn levels in the roots. *OsYSL15* appears to be regulated by a feedback mechanism that suppresses Fe transport when there is excess external Fe, whereas *OsIRT1* and *OsZIP1* are suppressed by the higher internal levels of Fe in the transgenic plants. This effect is partially (*OsIRT1*) or fully (*OsZIP1*) alleviated as the external Fe supply increases.

The transgenic plants generally accumulated more Fe and Zn in the flag leaf than wild-type controls, so it is likely that excess metals are sequestered into the vacuoles, thus reducing the amount available for seed loading. We therefore investigated the expression of endogenous genes encoding the Fe/Zn vacuolar transporter OsVIT1, the Fe-NA/Mn-NA phloem transporter OsYSL2, the Fe-regulated ZIP family Zn transporters OsZIP1 and OsZIP4, and the Fe storage proteins OsFERRITIN1/2. Compared with wild-type plants, *OsVIT1* was upregulated in the transgenic lines under all treatments and the expression levels mirrored the external Fe supply (Fig. 5). *OsYSL2* was expressed at similar levels in the transgenic and wild-type plants, and was expressed at the highest level under the double Fe feeding regime (Fig. 5). *OsFERRITIN1/2* and *OsZIP4* were upregulated in the transgenic lines under all three treatments. *OsZIP1* was expressed at higher levels in the transgenic plants under the normal and triple Fe treatments, but there was no difference between transgenic and wild-type lines under the double Fe treatment (Fig. 5). The higher levels of Fe and Zn in the flag leaf may induce the expression of vacuolar transporters to prevent excess seed loading with Fe and Zn. Accordingly, *OsVIT1* expression was regulated in response to the external supply of Fe and the abundance of Fe and Zn in the flag leaf, whereas *OsYSL2* appeared to limit the phloem transport of Fe. The stronger expression of *OsFERRITIN1/2* under all treatments indicates that Fe is stored in the chloroplast, thus reducing the availability of Fe for seed loading. The *OsZIP1*/*OsZIP4* expression profiles suggest a higher capacity for phloem loading Zn than Fe during the translocation of metals to the seed.

### Cd accumulation in the endosperm is inhibited by higher levels of Fe and Zn

**Fig. 6.**
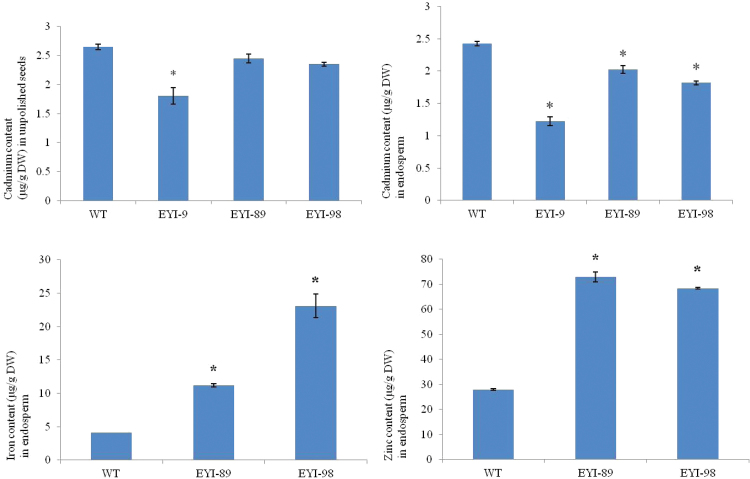
Levels of Cd (unpolished seeds and endosperm), Fe and Zn (endosperm) in wild-type (WT) rice and T_3_ transgenic lines co-expressing *OsNAS1* and *HvNAATb* (EYI-9, EYI-89, and EYI-98) grown in the presence of 100 µM FeCl_3_ and 10 µM CdCl_2_. Asterisks indicate a statistically significant difference between wild-type and transgenic plants as determined by Student’s *t*-test (*P*<0.05; *n*=6). The Cd content of the unpolished seeds and endosperm was significantly different in the wild-type and transgenic lines. Transgenic line EYI-9 did not produce enough seeds for analysis in the presence of Cd, so Fe and Zn levels were not measured in the endosperm. Seeds from the wild-type and transgenic lines were harvested at physiological maturity. (This figure is available in color at *JXB* online.)

T_3_ plants representing all three transgenic lines as well as wild-type controls were grown in the presence of 10 µM Cd and the T_4_ seeds were analysed to determine the metal content. The unpolished seeds of the transgenic lines accumulated 1.5-fold less Cd than wild-type seeds (Fig. 6). The wild-type endosperm contained ~9% less Cd than unpolished wild-type seeds, suggesting that >90% of the Cd in rice seeds accumulates in the endosperm. Interestingly, the endosperm of transgenic lines contained 14–18% less Cd than the unpolished transgenic seeds. Therefore, due to lower seed loading and endosperm retention of Cd, the transgenic endosperm accumulated 50% less Cd than the wild-type endosperm, possibly because the higher levels of Fe and Zn in the endosperm competitively inhibit Cd loading in the same tissue (Fig. 6). The levels of Fe and Zn in the transgenic endosperm were similar when plants were grown in the presence or absence of Cd, whereas the Zn content of wild-type endosperm decreased by 20% in the presence of Cd and the Fe content did not change (Figs 3 and 6).

The rice heavy-metal ATPase OsHMA2 and the low-affinity cation transporter OsLCT1 play key roles in Cd mobilization through the phloem and therefore regulate the subsequent accumulation of Cd in the seed. In immature T_4_ seeds, *OsHMA2* mRNA was expressed at similar levels in the wild-type and transgenic plants, whereas *OsLCT1* was suppressed in the transgenic lines (see Supplementary Fig. S3). These data suggest that seed loading with Cd was inhibited by two mechanisms acting in concert: first, by competition with the higher levels of Fe and Zn, and second, by the reduced capacity for Cd remobilization due to the loss of the corresponding transporter.

## Discussion

The uptake and distribution of metal ions in rice is controlled by NA and DMA, as well as metal transporters that recognize either free metal ions or metal–ligand complexes. One strategy to enhance Fe and Zn levels in rice endosperm is therefore to increase the NA and DMA content (Cheng *et al.*, 2007; Lee *et al.*, 2009*b*; Wirth *et al.*, 2009; Johnson *et al.*, 2011; Masuda *et al.*, 2012), but the mechanisms of metal homeostasis are complex and metal levels in the endosperm are limited by feedback control (Sperotto *et al.*, 2012*a*; Banakar *et al.*, 2017). Another complication is the promiscuous nature of metal ligands and transporters (Lee and An, 2009; Takahashi *et al.*, 2012; Nakanishi *et al.*, 2006), which means that the biofortification of endosperm with metal micronutrients may also drive the accumulation of toxic heavy metals such as Cd.

To investigate these issues we generated transgenic plants co-expressing two key enzymes in the phytosiderophore biosynthesis pathway (NAS and NAAT). The transgenic lines accumulated higher levels of NA and DMA in the roots, leaves, and seeds than wild-type plants. The best-performing lines accumulated 160-fold more NA and 29-fold more DMA than wild-type seeds, which far exceeds previous reports of up to 20-fold more NA and up to 5-fold more DMA based on the expression of individual *NAS* or *NAAT* transgenes (Takahashi *et al.*, 2001; Cheng *et al.*, 2007; Lee *et al.*, 2009*b*; Wirth *et al.*, 2009; Johnson *et al.*, 2011; Lee *et al.*, 2011; Masuda *et al.*, 2012). This indicates that the higher levels of NA and DMA in our transgenic lines reflect the co-expression of both transgenes.

Fe and Zn uptake and root-to-shoot translocation were more efficient in the transgenic plants than wild-type controls, leading to higher levels of Fe and Zn in the roots and leaves. Similar gains in Fe and Zn were previously achieved by mutating the *OsNAAT1* gene, causing an increase in NA levels (Cheng *et al.*, 2007), and by activation tagging the *OsNAS2* and *OsNAS3* genes, causing an increase in NA and DMA (Lee et al., 2009*b*, 2012). The T_2_ seeds of the best-performing transgenic lines accumulated up to 57 µg Fe g^−1^ DW (3.7-fold higher than wild-type) and up to 78 µg Zn g^−1^ DW (4.2-fold higher than wild-type) in the unpolished seeds. We observed positive correlation between the metal and phytosiderophore levels in the transgenic seeds (see Supplementary Fig. S4) as reported in previous studies (Supplementary Fig. S5) (Lee *et al.*, 2009*b*, 2011; Johnson *et al.*, 2011; Masuda *et al.*, 2012). After polishing, the transgenic endosperm contained up to 16 µg Fe g^−1^ DW and 65 µg Zn g^−1^ DW, each representing a 4-fold increase over wild-type. Similarly, the expression of *AtNAS1*, *HvNAS1*, and *OsNAS1–3* enhanced Fe and Zn loading in the endosperm (Wirth *et al.*, 2009; Johnson *et al.*, 2011; Masuda *et al.*, 2012), suggesting that the modulation of NA and DMA synthesis is a useful strategy to enhance the mineral content of rice endosperm.

Transgenic plants supplied with 200 µM Fe took up more Fe into the roots than wild-type plants, but when this was increased to 300 µM Fe the wild-type plants performed better. The high external concentration of Fe caused by the presence of DMA increases the concentration of Fe immediately above the root epidermis, resulting in the precipitation of Fe^3+^ due to the release of O_2_ from the roots (Chen *et al.*, 2006). The remobilization of Fe from the roots to the aboveground vegetative organs (culm and leaves) is necessary for seed loading (Sperotto *et al.*, 2012*b*). The culms and middle leaves of the transgenic plants generally accumulated more Fe than wild-type plants under all three treatments, suggesting that the root-to-shoot translocation of Fe remained effective.

Interestingly, the flag leaves of the transgenic plants generally accumulated more Fe than wild-type leaves, but there were no significant differences among the three treatments. Under Fe deficiency conditions, Fe remobilization from the middle leaf and flag leaf can contribute to Fe accumulation in the seeds, but this is not the case when there is a sufficient Fe supply (Fang *et al.*, 2008; Sperotto *et al.*, 2013; Sperotto, 2013). In *OsVIT1/2* knockdown mutants, the impaired vacuolar sequestration of Fe reduced the level of Fe in the flag leaves and higher levels accumulated in the seeds, suggesting that the flag leaf acts as a buffer to prevent seed overloading (Zhang *et al.*, 2012). The higher levels of Fe in the middle and flag leaves of our transgenic plants suggest that much of the Fe pool may have been sequestered into the vacuole and other organelles, making it unavailable for remobilization, as observed during the foliar application of Fe sprays to rice crops (Jin *et al.*, 2008) and when growing rice with a sufficient Fe supply (Sperotto *et al.*, 2013). Accordingly, we found that the accumulation of Fe in the endosperm of our transgenic plants fell within a similar range regardless of the feeding regime, and never exceeded 4.6-fold more than wild-type, revealing a homeostatic mechanism to prevent Fe overloading during seed development. This is consistent with the limited natural genetic diversity of Fe levels in rice, i.e. 2–5 µg g^−1^ DW (Sperotto *et al.*, 2012*a*). The threshold was 6 µg Fe g^−1^ DW in plants expressing *AtNAS1* (Wirth *et al.*, 2009), 14 µg Fe g^−1^ DW in plants expressing *OsNAS2* (Johnson *et al.*, 2011) and 22.5 µg Fe g^−1^ DW in our T_3_ seeds co-expressing *OsNAS1* and *HvNAATb*. Similarly, when six rice varieties were grown under conditions of acute and chronic Fe toxicity, the Fe content of the seeds did not change relative to control conditions in five of the varieties, whereas in the sixth variety 20% more Fe accumulated in the seeds under acute Fe toxicity conditions and 40% more accumulated under chronic Fe toxicity conditions (Frei *et al.*, 2016). These data indicate that Fe accumulation in seeds is genotype-dependent but homeostasis imposes a threshold for accumulation in all varieties to prevent Fe overloading, which could otherwise induce oxidative stress (Becker and Asch, 2005; Briat *et al.*, 2015). However, even with the Fe accumulation threshold in place, our transgenic lines accumulated a higher level of Fe (22.5 µg Fe g^−1^ DW) than the previous reports of 14 and 15 µg Fe g^−1^ DW (Johnson *et al.*, 2011; Trijatmiko *et al.*, 2016).

The amount of Zn taken up by the roots declined as the amount of Fe increased, suggesting competition for uptake through common transporters (Zhang *et al.*, 2012; Sperotto *et al.*, 2013). If the transporters show no preference, the lower uptake of Zn reflects statistical exclusion by the more abundant Fe^2+^ ions. At higher Fe levels, Fe precipitation on the root surface could also exclude Zn (Zhang *et al.*, 1998; Chen *et al.*, 2006). The amount of Zn in the culms and middle leaves of the transgenic plants was inversely related to the amount of Fe, suggesting a knock-on effect from the inhibition of Zn uptake into the roots. In the flag leaf, Zn generally accumulated to higher levels in the transgenic plants under all three treatments. Zn levels in the flag leaf may therefore also be regulated as a buffer system to limit Zn levels in the seed. The lowest levels of Zn in flag leaves were observed in plants supplied with 300 µM Fe, suggesting that Zn levels were affected even though the Fe content of the flag leaves remained similar across treatments. Metal homeostasis in different plant organs is interconnected (Jiang *et al.*, 2007; Fang *et al.*, 2008; Zhang *et al.*, 2012) so the varying Zn levels in the flag leaf under different treatments could in part reflect Zn levels in other tissues, which may be regulated by other Zn transporters (Ishimaru *et al.*, 2005; Yamaji *et al.*, 2013). Most of the Zn in the transgenic plants appears to be held in the culm, which acts as a ‘stopcock’ to regulate the flow of Zn to other tissues. This may operate through the sequestration of Zn into vacuoles by vacuolar Zn transporters such as MTP1 (Menguer *et al.*, 2013) and OZT1 (Lan *et al.*, 2013). Although our transgenic lines accumulated up to 84 µg Zn g^−1^ DW in the endosperm, there was no difference in Zn levels when varying the external Fe supply, suggesting that the buffering effect of the flag leaf may also act on Zn. In transgenic plants expressing *HvNAS1* alone, the higher levels of NA and DMA enhanced the remobilization of Zn from the husk to the seeds (Masuda *et al.*, 2009). Furthermore, seed loading is driven by the remobilization of Zn from tissues other than the roots and leaves (Yoneyama *et al.*, 2015). Therefore, even with the reduced Zn uptake and root-to-shoot translocation in the presence of high levels of external Fe, the higher levels of Zn in the endosperm of our transgenic lines suggest potential remobilization from tissues other than roots and leaves.

Metal uptake from the soil to the roots is controlled by the modulation of metal transporter genes (Ishimaru *et al.*, 2005; Chen *et al.*, 2008; Lee *et al.*, 2009*a*; Inoue *et al.*, 2009; Lee and An, 2009). We therefore investigated the expression of genes encoding the Fe^3+^–DMA transporter OsYSL15 (Inoue *et al.*, 2009; Lee *et al.*, 2009*a*), the Fe and Zn transporter OsIRT1 (Lee and An, 2009), and the Zn transporter *OsZIP1* (Chen *et al.*, 2008). Compared with wild-type plants, *OsYSL15* gene expression was induced in the roots of the transgenic lines in response to 100 and 200 µM Fe (highest at 100 µM Fe) but was suppressed in the presence of 300 µM Fe, which mirrors the level of Fe in roots. NA and DMA are important for the solubilization of Fe and Zn in soil (Cheng *et al.*, 2007; Nishiyama *et al.*, 2012; Suzuki *et al.*, 2008) so their accumulation in the transgenic lines promotes Fe and Zn solubilization in the nutrient solution and mobilization to the root surface. The 300 µM Fe treatment probably induces the formation of Fe plaques on the surface of the transgenic roots (inhibiting Fe uptake and suppressing *OsYSL15* expression) but not in wild-type roots (yielding the opposing expression profile). *OsIRT1* was expressed at lower levels in the transgenic plants than in wild-type plants at all Fe concentrations, but the gap between transgenic and wild-type plants declined as the Fe concentration increased. Furthermore, *OsIRT1* was induced more strongly in the transgenic lines under the double Fe feeding regime than the triple Fe feeding regime. Metal homeostasis involves complex mutual regulation by different metal transporters (Banakar *et al.*, 2017) so the expression level of *OsYSL15* may indirectly affect the expression of *OsIRT1* to regulate Fe uptake. *OsZIP1* was also induced at higher Fe levels, and the possible displacement of Zn by Fe at higher Fe levels at the root surface may also explain this trend.

The remobilization of Fe and Zn from vegetative tissues to seeds is important during grain filling, and is tightly regulated by metal transporters (Sperotto *et al.*, 2013). We investigated the expression of the vacuolar Fe transporter VIT1 (Zhang *et al.*, 2012), the Fe-NA/Mn-NA transporter YSL2 (Ishimaru *et al.*, 2010), and the Fe-regulated ZIP family transporters ZIP1 and ZIP4 (Ishimaru *et al.*, 2005; Chen *et al.*, 2008) in flag leaves. *OsVIT1* was upregulated in the transgenic lines under all treatments, mirroring the levels of Fe and Zn in the flag leaf. The overexpression of *OsVIT1* increased the vacuolar sequestration of Fe and Zn in the flag leaf, whereas the knockdown of the same gene enhanced Fe and Zn remobilization from the flag leaf and accumulation in seeds (Zhang *et al.*, 2012). The upregulation of *OsVIT1* in our transgenic lines therefore suggests a role for this transporter in the vacuolar sequestration of Fe and Zn. *OsYSL2* was expressed at similar levels in transgenic and wild-type plants under all treatments indicating that Fe remobilization from the flag leaf is limited. Ferritin is an iron storage protein located in the chloroplast. A single molecule of ferritin can store up to 4500 Fe atoms (Stein *et al.*, 2009). *OsFERRITIN1* and *OsFERRITIN2* encode two closely related isoforms of ferritin in rice (Stein *et al.*, 2009). In the flag leaf, *FERRITIN2* is expressed at higher levels than *FERRITIN1* (Stein *et al.*, 2009; Paul *et al.*, 2012). Therefore, *OsFERRITIN2* in the flag leaf may be upregulated in our transgenic lines under all three Fe treatment regimes, suggesting that Fe is sequestrated in the chloroplast and unavailable for phloem loading and translocation to the seed. *OsZIP4* was upregulated in the transgenic lines under all treatments, reflecting the lower level of Zn compared with Fe in the flag leaf and suggesting a preference for Zn over Fe during endosperm loading in the transgenic lines.

NA and DMA regulate the accumulation of Fe, Zn, Mn, and Cu in rice (Masuda *et al.*, 2012) but they also have a low affinity for Cd, which is detrimental to human health (Meda *et al.*, 2007; Cheng *et al.*, 2007). Our transgenic plants grown in the presence of Cd accumulated 1.5-fold and 2-fold lower levels of this heavy metal in the unpolished seeds and endosperm, respectively. The endosperm is the preferred site for both Zn and Cd accumulation (Yang *et al.*, 1998; Lee *et al.*, 2011; Sperotto *et al.*, 2012*b*; Takahashi *et al.*, 2012; Uraguchi and Fujiwara, 2012; Yoneyama *et al.*, 2015) suggesting that strategies to increase Fe and/or Zn accumulation may inhibit Cd loading in the seeds. Accordingly, Cd levels in seeds can be reduced by using Zn fertilizers (Oliver *et al.*, 1994; Sarwar *et al.*, 2015) and external Fe supplements (Hassan *et al.*, 2005; Shao *et al.*, 2007), whereas Fe limitation increases Cd levels (Nakanishi *et al.*, 2006). The heavy metal ATPase OsHMA2 and low-affinity cation transporter OsLCT1 facilitate seed loading with Cd (Ueno *et al.*, 2010; Uraguchi *et al.*, 2011; Yamaji *et al.*, 2013). *OsHMA2* mRNA levels were similar in the wild-type and transgenic lines, whereas *OsLCT1* mRNA was depleted in the transgenic plants, mirroring the lower levels of Cd in the endosperm. The loss of Cd may therefore reflect the preferential loading of Fe and Zn in the endosperm (Basnet *et al.*, 2014) as well as the suppression of *OsLCT1* expression (Uraguchi *et al.*, 2011).

**Fig. 7.**
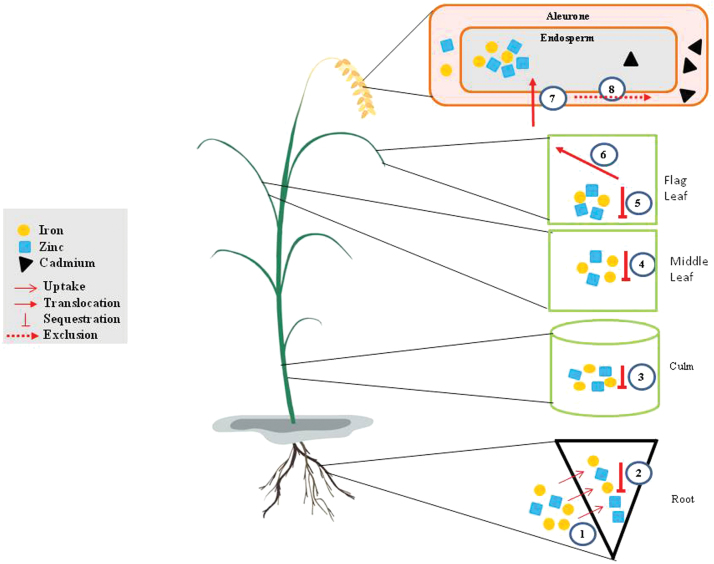
A mechanistic model of iron (Fe) and zinc (Zn) homeostasis in rice endosperm, explaining the presence of an upper limit for Fe and Zn accumulation. Fe and Zn uptake in the roots is regulated by modulating endogenous metal transporter expression in response to Fe levels (1), by sequestering Fe/Zn in the roots (2), culm (3), middle leaf (4), and flag leaf (5), and by controlling phloem Fe/Zn remobilization from the flag leaf to the seeds by the modulation of metal transporter expression in these tissues (6), particularly vacuolar transporters in the flag leaf to promote vacuolar sequestration (5). The accumulation of Fe and Zn in the endosperm can therefore increase by a maximum of 4-fold when phytosiderophores are not limiting (7) but this is sufficient to competitively inhibit the Cd loading in the endosperm, causing this toxic metal to accumulate in the bran instead (8).

Our data suggest a model for Fe and Zn homeostasis in the endosperm and the competitive inhibition of Cd accumulation as summarized in Fig. 7. The upper limit for Fe and Zn in the endosperm is imposed by their sequestration in the roots, culm, middle leaf, and flag leaf. This is principally achieved by regulating metal uptake into the roots and the vacuolar sequestration of metals in the flag leaf, in each case by modulating the expression of endogenous metal transporters. Despite the upper limit for Fe and Zn accumulation in the endosperm, higher levels of Fe and Zn can competitively inhibit the accumulation of Cd.

In conclusion, we have shown that the co-expression of *OsNAS1* and *HvNAATb* in rice increases the abundance of NA and DMA and allows the accumulation of higher levels of Fe and Zn in the endosperm. Increasing the external supply of Fe affected the uptake of Fe and Zn into the roots and the mobilization of these metals in the aboveground organs, but compensatory mechanisms involving vacuolar sequestration in the flag leaf have a buffering effect and impose strict limits on the accumulation of metal micronutrients in the endosperm, resulting in maxima of 22.5 and 84 µg g^−1^ DW for Fe and Zn, respectively. Furthermore, the preferential retention of Fe and Zn in the endosperm led to the competitive exclusion of Cd, halving the amount of Cd in the endosperm. This can provide a useful strategy to increase the abundance of metal nutrients in rice endosperm while ensuring that toxic metals are exported to the bran. Such strategies could help simultaneously to address micronutrient deficiency and heavy metal toxicity in communities that rely predominantly on cereal-based diets.

## Supplementary data

Supplementary data are available at *JXB* online.

Fig. S1. Fe and Zn content of roots and leaves of wild-type and transgenic rice plants.

Fig. S2. Height of wild-type and transgenic plants grown under different Fe feeding regimes.

Fig. S3. Quantitative real-time RT-PCR analysis of *OsHMA2* and *OsLCT1*.

Fig. S4. Scatter graph and linear correlations between NA and/or DMA levels and Fe and Zn levels.

Fig. S5. Scatter graph and linear correlations between unpolished grain NA and/or DMA levels and Fe and Zn levels.

Table S1. Genes and primers for cloning and quantitative real-time RT-PCR analysis.

Table S2. Fe and Zn content of unpolished wild-type and transgenic rice seeds.

## Author contributions

RB, AAF, and PC designed the research; RB performed the experiments; RB and AAF analysed the data; RB, AAF, PDB, JA, TC, and PC wrote the paper.

## Competing financial interests

Authors declare no conflict of financial interest.

## Supplementary Material

Supplementary_Figures_S1_S5_Tables_S1_S2
