# Supplementary material for: Phytosiderophores determine thresholds for iron and zinc accumulation in biofortified rice endosperm while inhibiting the accumulation of cadmium
Source: J Exp Bot. 2017 Sep 28;68(17):4983–95. doi: 10.1093/jxb/erx304 (PMC5853871; doi:10.1093/jxb/erx304)
Supplement: Supplementary_Figures_S1_S5_Tables_S1_S2 [file erx304_suppl_supplementary_figures_s1_s5_tables_s1_s2.pdf]

Phytosiderophores determine thresholds for iron and zinc accumulation in biofortified rice endosperm while inhibiting accumulation of cadmium

Raviraj Banakar[1], Ana Alvarez Fernandez[2], Pablo Díaz-Benito[2], Javier Abadia[2], Teresa Capell[1], Paul Christou[1, 3]*

[1]*Departament de Producció Vegetal i Ciència Forestal, Universitat de Lleida-Agrotecnio Center Lleida, Spain*

[2]*Department of Plant Nutrition, Estación Experimental de Aula Dei, Consejo Superior de Investigaciones Científicas (CSIC), P.O. BOX 13034, E-50080 Zaragoza, Spain*

[3] *ICREA, Catalan Institute for Research and Advanced Studies, Passeig Lluís Companys 23, 08010 Barcelona, Spain*

Email Addresses of Authors:

ravirajbanakar@pvcf.udl.cat

ana.alvarez@eead.csic.es

pablodiazbenito@eead.csic.es

jabadia@eead.csic.es

teresa.capell@pvcf.udl.cat

Corresponding author contact details:

*Paul Christou

Departament de Producció Vegetal i Ciència Forestal

Universitat de Lleida-Agrtotecnio Center Lleida

Av Alcalde Rovira Roure 191, Lleida, Spain.

Fax +34 973 70 29 24

E-mail: christou@pvcf.udl.es

Supporting information

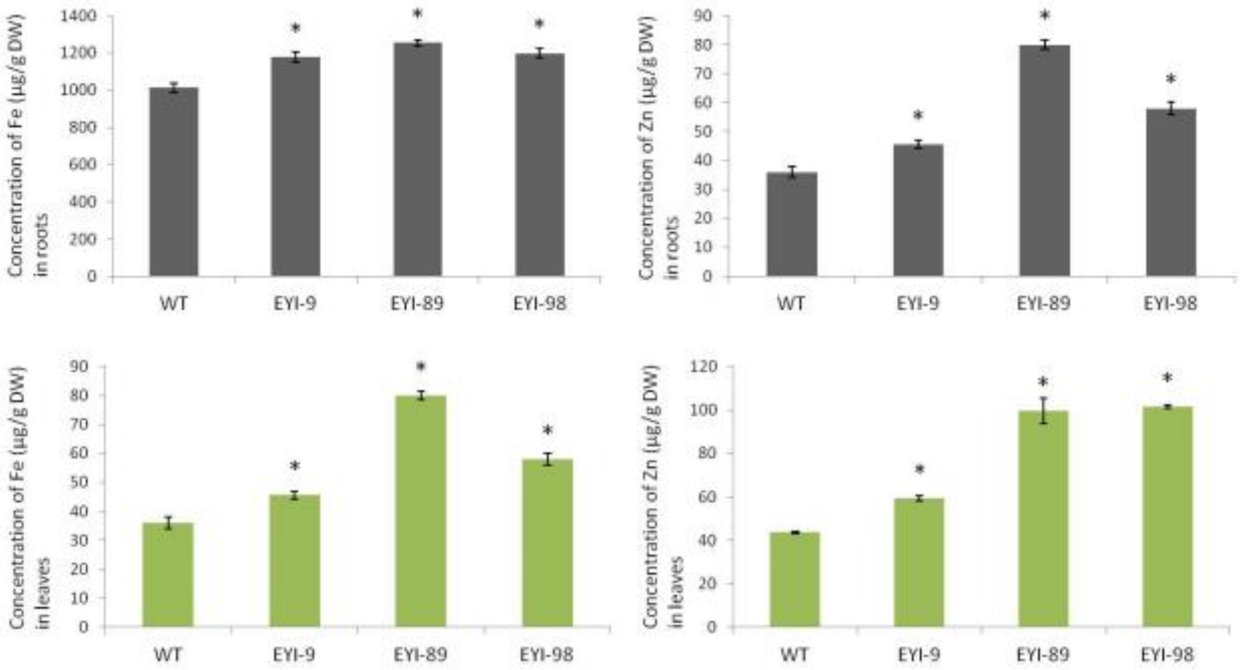

Fig. S1. Fe and Zn content (µg g^{-1} DW) in roots and leaves of wild-type (WT) rice and three different T_1 transgenic lines co-expressing *OsNAS1* and *HvNAATb* (EYI-9, EYI-89 and EYI-98). DW = dry weight. Asterisks indicate a statistically significant difference between wild-type and transgenic plants as determined by Student's *t*-test ($p < 0.05$; n = 6). Roots and leaves from the wild-type and transgenic lines were harvested at the vegetative stage.

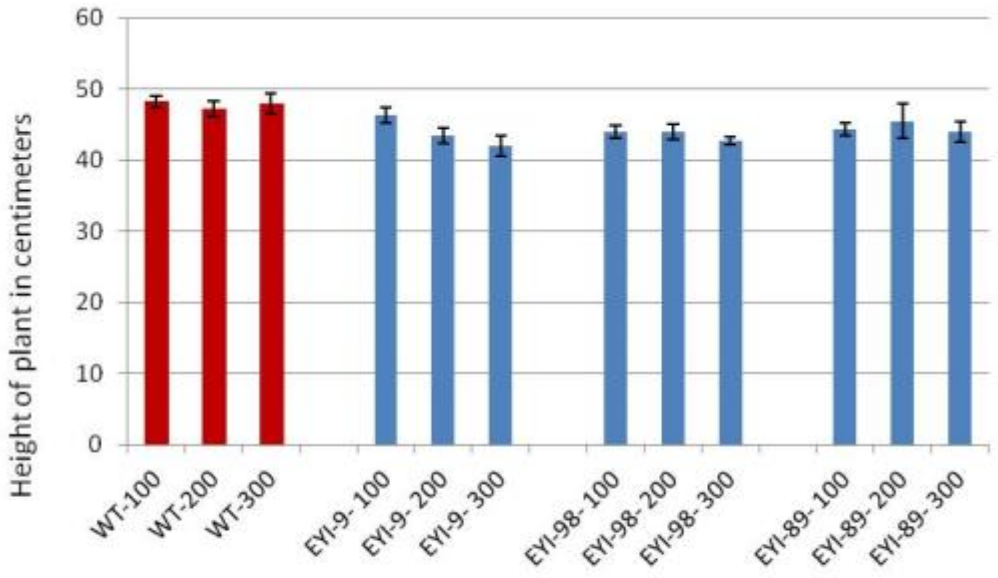

Fig. S2. Height of wild type and T_2 transgenic plants co-expressing *OsNAS1* and *HvNAATb* (EYI-9, EYI-89 and EYI-98). Data are means (n = 6).

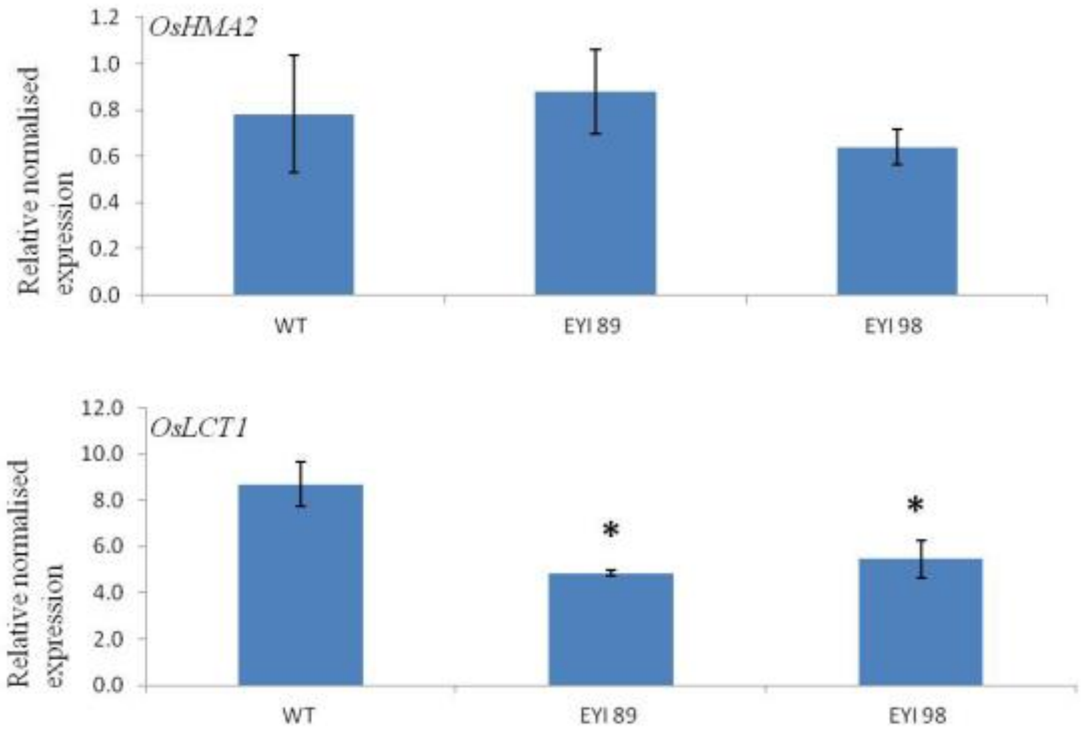

Fig. S3. Quantitative real-time RT-PCR analysis of *OsHMA2* and *OsLCT1* following the treatment of wild-type (WT) plants and T_3 transgenic lines co-expressing *OsNAS1* and *HvNAATb* (EYI-89, EYI-98) with 10 mM Cd at the grain-filling stage. Each value is the

mean of three independent experiments ± SE. Relative transcript levels are normalized against *OsACTIN1*. Asterisks indicate a statistically significant difference between wild-type and transgenic plants as determined by Student's *t*-test ($p<0.05$; n = 3). Gene-specific primers are listed in Supplementary Table S2. Both the wild-type and transgenic lines were grown under nutrient sufficient conditions, and the seeds were harvested at the grain-filling stage for total RNA isolation.

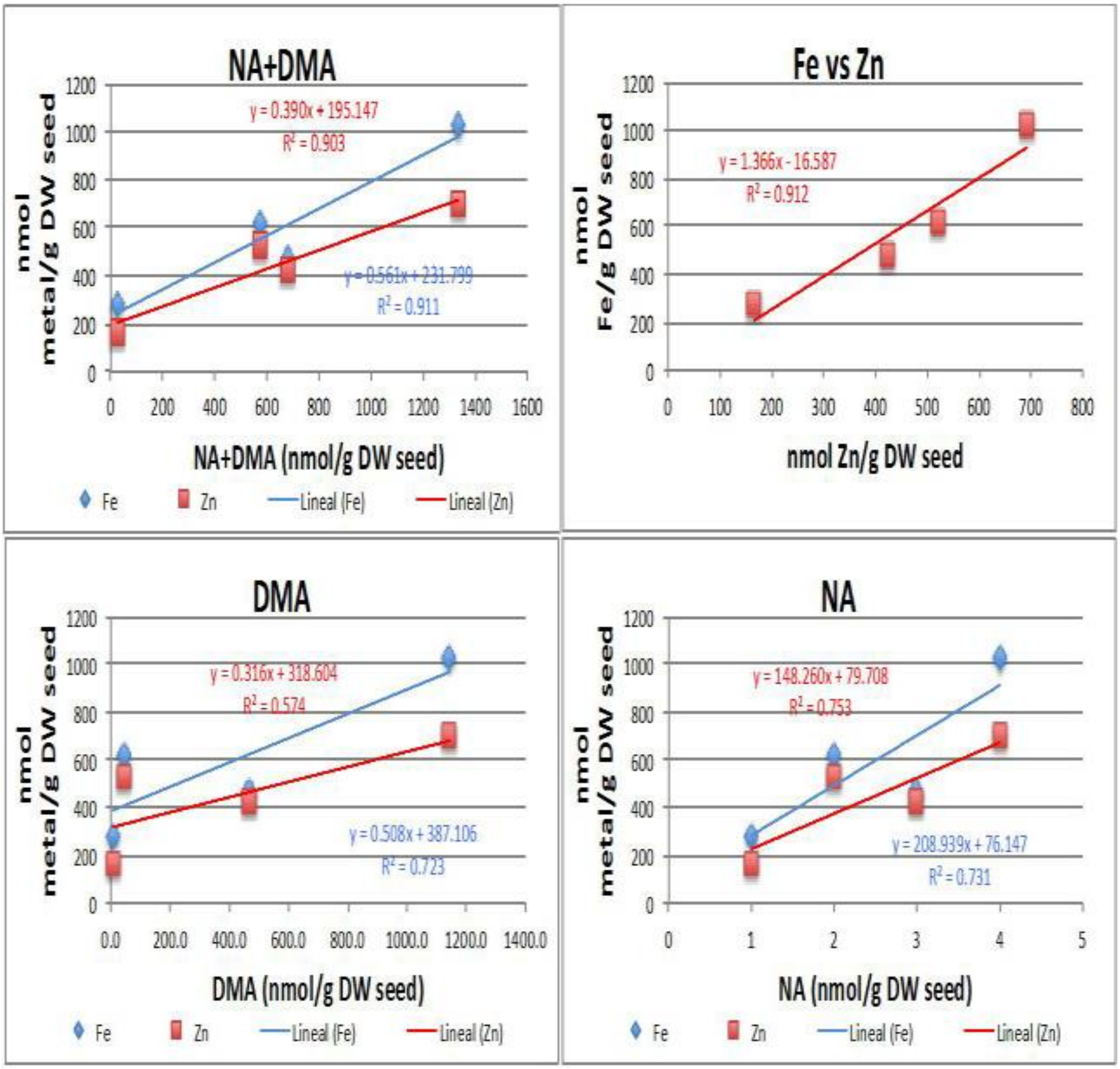

Fig. S4. Scatter graph and linear correlations between NA and/or DMA levels and Fe (blue diamonds) and Zn (red squares) levels in the unpolished seeds of wild-type plants and T_2 transgenic lines co-expressing *OsNAS1* and *HvNAATb* (EYI-9, EYI-89 and EYI-98).

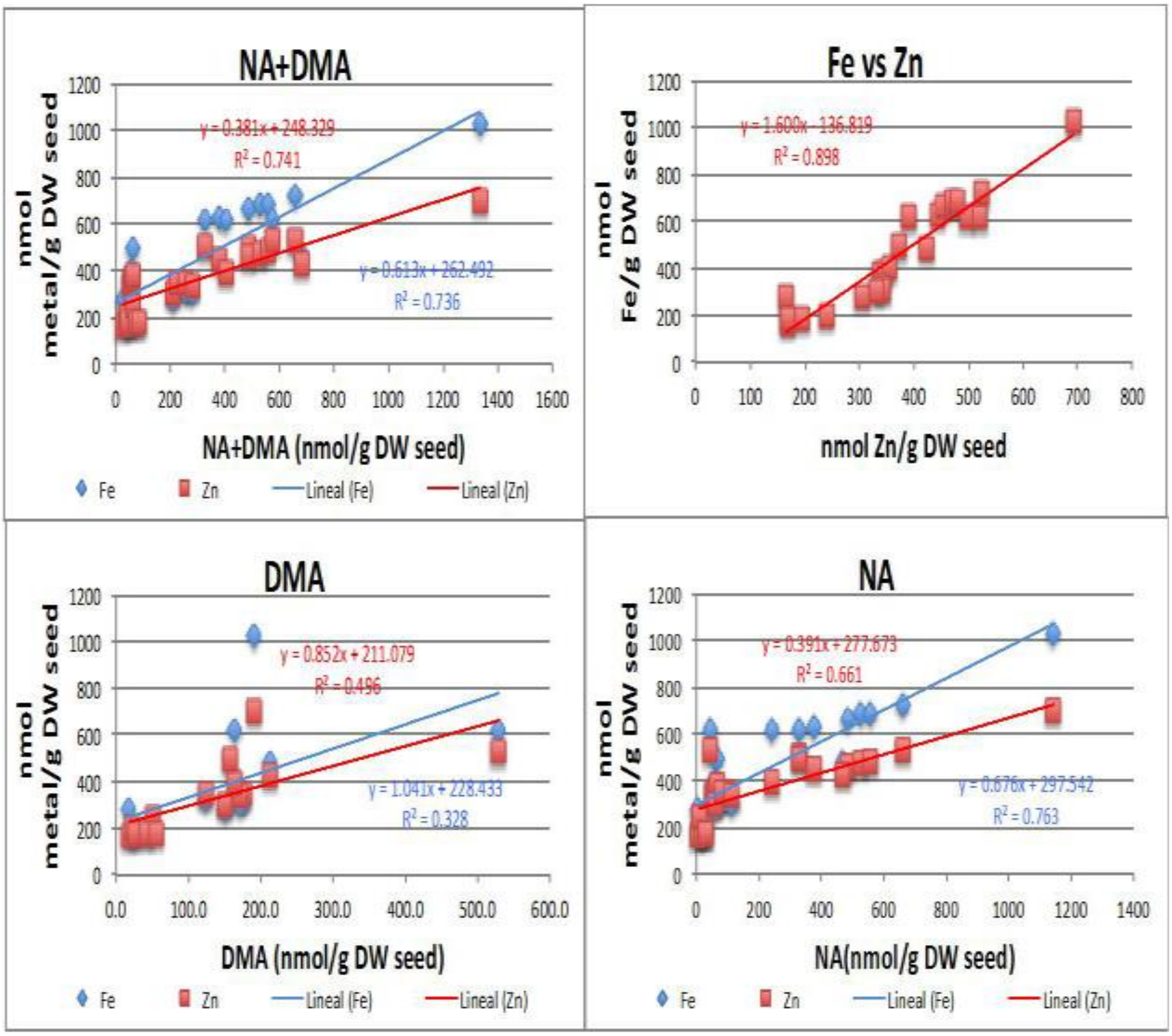

Fig. S5. Scatter graph and linear correlation between unpolished grain NA and/or DMA levels and Fe (blue diamonds) and Zn (red square) levels for the genotypes in Figure 2 and for those reported by others (Lee *et al*., 2009; Johnson *et al*., 2011; Lee *et al*., 2011; Masuda *et al*., 2012).

Gene	GenBank ID	Amplicon (bp)	Forward primer	Reverse primer
Primers for cloning				
OsNAS1	AB021746.2	999 bp	*OsNAS1*-BamHI-FOR<br>AGG ATC CAT GGA GGC TCA GAA CCA AGA GGT CG	*OsNAS1*-HindIII-REV<br>AAA GCT TCA TAA TAT AGT GCG CCT TTC GAT CGT CCG GCT GT
HvNAATb	AB005788.1	1656 bp	*HvNAATb*-BamHI-FOR<br>AGG ATC CAT GGC CAC CGT ACG GCC AGA GAG CGA CG	*HvNAATb*-HindIII-REV<br>AAA GCT TCT AGC AAT CAT CGC TCG CTC GAA TTT CTC
Primers for real-time RT-PCR				
OsYSL15	AB190923.1	78	CTGGTGCTGTTTGCTTGGA	ATGAGCCCAGACGCAACAGC
OsIRT1	BAB85123.1	139	GCATCATGCAATTCGCTGC	CCTGAACAACCACGCTACAA
OsZIP1	AY324148.1	90	CGATGGTCCTCTTCTTCTGC	GTCGGGCTGCTCTCGTTGTA
OsZIP4	AB126089.1	107	TCCACCAGTTCTTTGAAGGC	CGGAGCAGTGAGGGAGAAGA
OsVIT1	NM_001059545.1	107	CCACAGCACAGAACGCCAT	GCTGAGGAATGGACGGTTT
OsYSL2	AB164646	169	GGTGGAGAGAGTTGTGGGTT	TCATTCCCGCACCAACATA
OsFERRITIN1/ OsFERRITIN2	AF519570.1/ AF519571.1	132	GCTTGAATGGAGGAGACTGTG	CTCCATTGCTACTGCGTGCT
OsLCT1	AB905364.1	100	GCTCAAGTGGATGGCGGACA	GCCACCATCTTGTCCTTGCTT
OsHMA2	AB697186.1	126	CGAGGAATACACCCGCATCAGC	CAAGCAGAGCCACGAAAAGAGC
OsACTIN1	AB047313.1	120	TCA TGT CCC TCA CAA TTT CC	GAC TCT GGT GAT GGT GTC AGC

Table S1. Genes and primers for cloning and quantitative real time RT-PCR analysis.

Line	Fe in unpolished seeds (μg/g DW)	Zn in unpolished seeds (μg/g DW)
EYI-9	26.35 *	47.49*
EYI-89	56.68*	77.80*
EYI-98	34.47*	58.7*
EYI-0.7	33*	43*
EYI-0.2	30*	42*
EYI-38	25*	37*
EYI-4	26.29*	28.02*
EYI-8	22.96*	21.57*
EYI-12	23.42*	23.24*
EYI-121	21.92*	22.77*
WT	15.33	18.51

Table S2. Fe and Zn content of unpolished seeds from wild-type (WT) and T_2 transgenic rice plants. DW = dry weight. Ten independent transgenic lines co-expressing *OsNAS1* and *HvNAATb* are shown. Asterisks indicate a statistically significant difference between wild-type and transgenic plants as determined by Student's *t*-test ($p<0.05$; n = 6). Both the WT and transgenic lines were grown in nutrient sufficient conditions, and the seeds were harvested at physiological maturity.
